# Comparative Genome Analysis of *Enterobacter cloacae*

**DOI:** 10.1371/journal.pone.0074487

**Published:** 2013-09-12

**Authors:** Wing-Yee Liu, Chi-Fat Wong, Karl Ming-Kar Chung, Jing-Wei Jiang, Frederick Chi-Ching Leung

**Affiliations:** 1 School of Biological Sciences, the University of Hong Kong, Hong Kong SAR, People’s Republic of China; 2 Bioinformatics Centre, Nanjing Agricultural University, Nanjing, China; Beijing Institute of Genomics, China

## Abstract

The *Enterobacter cloacae* species includes an extremely diverse group of bacteria that are associated with plants, soil and humans. Publication of the complete genome sequence of the plant growth-promoting endophytic *E. cloacae* subsp. *cloacae* ENHKU01 provided an opportunity to perform the first comparative genome analysis between strains of this dynamic species. Examination of the pan-genome of *E. cloacae* showed that the conserved core genome retains the general physiological and survival genes of the species, while genomic factors in plasmids and variable regions determine the virulence of the human pathogenic *E. cloacae* strain; additionally, the diversity of fimbriae contributes to variation in colonization and host determination of different *E. cloacae* strains. Comparative genome analysis further illustrated that *E. cloacae* strains possess multiple mechanisms for antagonistic action against other microorganisms, which involve the production of siderophores and various antimicrobial compounds, such as bacteriocins, chitinases and antibiotic resistance proteins. The presence of Type VI secretion systems is expected to provide further fitness advantages for *E. cloacae* in microbial competition, thus allowing it to survive in different environments. Competition assays were performed to support our observations in genomic analysis, where *E. cloacae* subsp. *cloacae* ENHKU01 demonstrated antagonistic activities against a wide range of plant pathogenic fungal and bacterial species.

## Introduction

*Enterobacter cloacae* is a gram-negative Proteobacterium belonging to the Enterobacteriaceae family. Within this family, *Enterobacter* is most closely related to, and is grouped in a sub-clade with, *Klebsiella* . The two groups of bacteria diverged from a sub-clade consisting of *Escherichia* , *Citrobacter* and *Salmonella* [1].

The *E. cloacae* species comprises an extremely diverse group of bacteria that has been found in diverse environments, ranging from plants to soil to humans. Plant pathogenic strains of *E. cloacae* have been reported to cause *Enterobacter* bulb decay in onion plants and bacterial wilt in mulberry [2,3]; endophytic *E. cloacae* strains have been shown to colonize and benefit plant growth in various crops, such as soybean, cucumber, corn, rice and ginger [4,5]. Previous biological studies of several plant-origin isolates have shown that *E. cloacae* has antagonistic effects against the oomycete pathogen *Pythium ultimum* [6], the fungal pathogens *Fusarium* *moniliforme* and *Fusarium oxysporum* [5,7] and the bacterial pathogen *Ralstonia solanacearum* [8]. Additionally, several strains of *E. cloacae* are considered to be plant growth-promoting rhizobacteria (PGPR). The genomic features that underlie the antagonistic characteristics have been demonstrated in previous genome analyses of model PGPR species, such as the gram-negative *Pseudomonas* and the gram-positive *Bacillus* [9,10,11], but these features have not been studied in *Enterobacter* spp.

*E. cloacae* is best known as a human opportunistic pathogen that is commonly found in hospitals and causes a wide range of infections, such as lower respiratory tract infections, urinary tract infections and meningitis [12]. Outbreaks usually occur in Intensive Care Units, primarily affecting patients in vulnerable age groups and patients who are hospitalized for a prolonged period. *E. cloacae* is clinically significant, particularly because its strains usually carry multiple antibiotic resistance genes [13,14]. The complete genome sequence was published for *E. cloacae* subsp. *cloacae* ATCC13047, a classic strain isolated in the last century from human brain fluids, but no detailed analysis or supporting data were reported [15].

Compared with other genera in the family of Enterobacteriaceae such as *Escherichia* , *Salmonella* and *Yesinia*, which are well-known for their association with pathogenicity in humans and animals, there is scarcity of genomic data for *Enterobacter* . Eight complete *Enterobacter* genomes have been reported to NCBI (http://www.ncbi.nlm.nih.gov/genome/1219): *E. aerogenes* KCTC2190, *E. cloacae* subsp. *cloacae* ATCC13047, *E. cloacae* subsp. *dissolvens* SDM, *E. cloacae* subsp. *cloacae* NCTC9394, *E* *. lignolyticus* SCF1 (named as *E. cloacae* SCF1 in NCBI database), *E. asburiae* LF7a, *E. cloacae* EcWSU1 and *Enterobacter* sp. 638. These genome sequencing projects mostly emphasized the potential for the use of *Enterobacter* in industrial applications, such as lignin degradation [1,16] and 2,3-butanediol production [17,18,19]. A detailed genome study illustrated the synergistic interactions between the poplar tree host and the growth promoting endophyte *Enterobacter* sp. 638 [18]. Four of the available *Enterobacter* genomes belong to *E. cloacae*, which include human opportunistic pathogens *E. cloacae* subsp. *cloacae* NCTC9394 and *E. cloacae* subsp. *cloacae* ATCC13047 [15], a plant pathogen *E. cloacae* EcWSU1 [20] and a 2,3-butanediol producing *E. cloacae* subsp. *dissolvens* SDM [19].

Using the complete genome sequence of the plant growth-promoting endophytic *E. cloacae* subsp. *cloacae* ENHKU01 and the available complete genome sequence data from different strains of *E. cloacae* [15,19,20,21], we have performed a comparative genome analysis between *E. cloacae* strains that originated from diverse environments. In this study, we identified crucial conserved genomic factors that support the antagonistic functions of *E. cloacae*. Genomic factors differentiating the various strains of *E. cloacae* were also investigated. Also, this study is the first comprehensive comparative genome analysis for *E. cloacae* genomes.

## Materials and Methods

### Discovery of *E. cloacae* subsp. *cloacae* ENHKU01

*E. cloacae* subsp. *cloacae* ENHKU01 was isolated from a pepper plant infected by *R. solanacearum* in Hong Kong in May 2010. Three single colony subcultures were performed to obtain a pure isolate on TTC (2,3,5-triphenyl- tetrazolium chloride) medium w/v 1.5% agar plates at 28°C. An inoculation and re-isolation experiment using 4-week old tomato and pepper seedlings was carried out to confirm its endophytic characteristic in plants. Unlike *R. solanacearum*, the bacterial isolate did not cause wilting symptoms in these plants following inoculation. Inoculation on potato tubers and onion bulbs also indicated that the isolate did not cause disease in these plants. Sequencing and BLAST analysis of partial 16S rRNA and housekeeping genes (*fusA*, *gyrB*, *hsp60*, *rpoB*) [22] was conducted for classification, and the bacterial strain was shown to belong to *E. cloacae* and was most closely related to *E. cloacae* subsp. *cloacae* ATCC13047.

### Genome sequencing and comparative analysis

Whole genome sequencing and annotation of *E. cloacae* subsp. *cloacae* ENHKU01 was performed as described in Liu et al., 2012 [21]. In brief, *de novo* shotgun sequencing and paired-end sequencing strategies were applied to produce the whole genome sequence of ENHKU01 using the 454 GS Junior platform (454 Life Sciences, Branford, CT, USA). Newbler Assembler software (454 Life Sciences, Branford, CT, USA) was used to construct a draft genome with one scaffold containing 36 contigs [23]. To complete the whole-genome sequence of *E. cloacae* subsp. *cloacae* ENHKU01, sequence gaps were filled by PCR, primer walking and Sanger sequencing.

Gene annotation and analysis was performed using NCBI Prokaryotic Genomes Automatic Annotation Pipeline (PGAAP) [24]. Annotation was performed using BLASTP, and the protein sequences of predicted genes were searched against all proteins from complete microbial genomes and aligned with the best BLAST-hit [25]. Genome sequence data have been deposited to NCBI, GenBank accession number is CP003737.1, and it is available for download at NCBI: ftp://ftp.ncbi.nlm.nih.gov/genomes/Bacteria/Enterobacter_cloacae_ENHKU01_uid172463/.

For comparative genome analysis, complete genome sequence data of *Enterobacter* species: *E. aerogenes* KCTC2190 (GenBank accession CP002824), *E. cloacae* subsp. *cloacae* ATCC13047 (GenBank accession CP001918, CP001919 and CP001920), *E. cloacae* subsp. *dissolvens* SDM (GenBank accession CP003678), *E* *. lignolyticus* SCF1 (named as *E. cloacae* SCF1 in NCBI database) (GenBank accession CP002272), *E. asburiae* LF7a (CP003026, CP003027 and CP003028), *E. cloacae* EcWSU1 (GenBank accession CP002886 and CP002887) and *Enterobacter* sp. 638 (GenBank accession CP000653 and CP000654) were obtained from NCBI (ftp://ftp.ncbi.nlm.nih.gov/genomes/Bacteria/). *E. cloacae* subsp. *cloacae* NCTC9394 sequence data were not available for download; thus, it was not included in this study. Scaffolds of the *Enterobacter* species involved in this study were also uploaded to the Rapid Annotations using Subsystems Technology (RAST) server for SEED-based automated annotation, whole-genome sequence-based comparative analysis and Kyoto Encyclopedia of Genes and Genomes (KEGG) metabolic pathway for comparative analysis [26]. Efficient Database framework for comparative Genome Analyses using BLAST score Ratios (EDGAR) was used for core genome, pan genome and singleton analysis, and Venn diagram construction using *E. cloacae* subsp. *cloacae* ENHKU01 as a reference genome [27]. Further comparative analysis was performed for specific regions and genes-of-interest by BLASTN, BLASTX and BLASTP.

### Phylogenomic analysis of *Enterobacter* spp

An in-house pipeline has been developed for the phylogenomic analysis of *Enterobacter* spp. Scaffolds and genome data of the chromosome of eight *Enterobacter* genomes and three *Pantoea* genomes: *Pantoea* sp. At-9b (NC_014837), *P* *. vagans* C9-1 (NC_014562) and *P* *. ananatis* LMG20103 (NC_013956), were downloaded from NCBI (ftp://ftp.ncbi.nlm.nih.gov/genomes/). Gene clustering was performed using OrthoMCL 2.0 under default parameters [28]. A minimum length coverage filter of 85% was applied to further confirm orthology and the families that passed were aligned by MUSCLE v3.8.31 [29]. The corresponding amino acid alignments of 1732 core genes were created and then concatenated to construct a “supergene” for the reconstruction of the phylogenomic tree of *Enterobacter* spp. by MrBayes v3.2 using the WAG model. Ten million generations were performed with four chains; burn-in was set to 10,000 generations [30].

Phylogenetic analysis. To analyze the phylogeny of specific genes-of-interest, partial- or full-length CDS of *E. cloacae* subsp. *cloacae* ENHKU01 was used as a bait to search for orthologs in the NCBI database by BLASTP/ BLASTX using an E-value cut-off of 1e-10. Protein sequences of the top 100 hits were obtained. MEGA5 was used to perform phylogenetic analysis, multiple alignments of protein sequences were built by CLUSTALW and a 100 bootstrap replicate Neighbor-joining tree was constructed for phylogenetic analysis [31].

Phylogenetic analysis of the Type VI Secretion System (T6SS) was performed using a similar strategy as described in previous studies with modifications [32]. T6SS component genes were searched in the Integrated Microbial Genome database version IMG 3.4 in Joint Genome Institute (http://img.jgi.doe.gov) with 1350 finished bacterial genomes. T6SS clusters containing core component genes COG0542 (*clpV*) with at least four of the following loci: COG3516 (ImpB), COG3517 (ImpC), COG3519 (VasA), COG3520 (VasB) and COG3522 (ImpJ/VasE) were included for further analysis. T6SS clusters with fewer than five core component genes were discarded. As a result, a total of 346 T6SS clusters were identified in 230 bacterial genomes, mostly belonging to the five subdivisions of Proteobacteria. Two T6SSs of ENHKU01 were manually identified and reconfirmed by BLAST and included in the analysis. MEGA5 was used to perform phylogenetic analysis for the ClpV orthologs in a total of 348 T6SS [31]. Multiple alignments of ClpV protein sequences were built by CLUSTALW, and a Neighbor-joining tree was constructed for evolutionary analysis.

### Isolation of other bacterial, fungal isolates and antagonistic bioassays

Bioassays were designed to investigate the antagonistic activities of *E. cloacae* subsp. *cloacae* ENHKU01 against various plant bacterial and fungal pathogens. All biological samples involved in this study were collected within a 200 km zone in Hong Kong SAR and Guangdong, China. Plant pathogenic fungi *Alternaria* sp., *Choanephorainfundibulifera* , *Colletotrichum* *capsici* , *Didymellabryoniae* *, Fusarium oxysporum*, *Sclerotinia sclerotiorum* and *Sclerotinia rolfsii* were isolated from infected host plants and purified by subsequent subculture on potato dextrose agar (PDA) plates at 28°C; next, pure isolates were obtained and strain identification was confirmed by cloning and sequencing (Clover Seed Co., Ltd., Hong Kong). For fungal antagonistic assays, each challenging fungus was grown for 4 days at 28°C. A sample composed of 0.5 cm of agar with hyphae was cut and placed at 2 cm from the edge of a new potato dextrose agar (PDA) plate. An overnight culture of ENHKU01 was streaked across the middle of the plate [33]. Controls were set up for challenging fungus without *E. cloacae* subsp. *cloacae* ENHKU01. Fungal growth was monitored for two weeks. Three independent experiments were performed for each *E. cloacae* subsp. *cloacae* ENHKU01-fungal antagonistic assay.

The bacterial isolate, *Ralstonia solanacearum*, was isolated from the same pepper plant as the *E. cloacae* subsp. *cloacae* ENHKU01 isolate. Three single-colony subcultures were performed to obtain a pure isolate on TTC medium w/v 1.5% agar plates at 28°C. Identification of *R. solanacearum* was confirmed by cloning and sequencing of partial 16S rRNA and fulfilling Koch’s Postulates (Clover Seed Co., Ltd., Hong Kong) [34]. *E. cloacae* subsp. *cloacae* ENHKU01- *R. solanacearum* competition assays were performed in both planktonic and biofilm cultures. To prepare for the competition assay, bacterial cultures were grown overnight in TTC medium and adjusted to OD_600_=0.1(~1 x 10^8^ c.f.u/ml). For the planktonic culture, equal volumes (25 ml) of ENHKU01 and *R. solanacearum* were mixed in a 1:1 ratio in a 250 ml flask and incubated in a shaking incubator (250rpm at 28°C). One milliliter of culture was collected at 2, 4, 6 and 24 hours after incubation for quantification. For the biofilm culture, ENHKU01 and *R. solanacearum* were mixed in a 1:10 ratio. 20 µl of ENHKU01 culture was placed on a TTC w/v 1.5% agar plate and incubated at 28°C. Biofilm samples were collected for quantification using 1 ml pipette tips and re-suspended in water after 24 hours of incubation. Controls consisted of samples containing only ENHKU01 or *R. solanacearum* for both planktonic and biofilm competition assays. For quantification (including initial concentration of ENHKU01 and *R. solanacearum*), serial dilutions were prepared for each sample to a desirable concentration and plated on TTC w/v 1.5% agar. C.F.U.s were counted after incubation at 28°C for 48 hours. ENHKU01 and *R. solanacearum* can be distinguished by morphological differences in size, color and texture. Four replications were performed for each assay.

## Results and Discussion

### Phylogenomic analysis of *Enterobacter*

**Figure 1:**
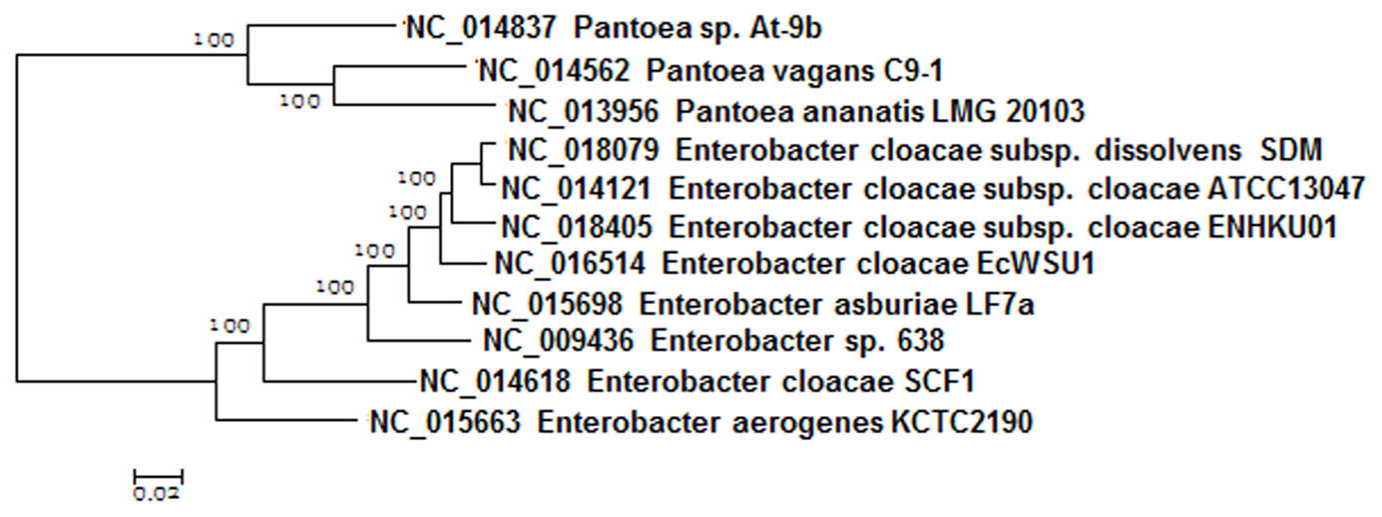
Phylogenomic analysis of *Enterobacter* spp. Bayesian tree with posterior p-values of the genomes of *Enterobacter* spp. using 1732 core genes of eight *Enterobacter* and three *Pantoea* genomes.

The classification of *Enterobacter* spp. using 16S rRNA and house-keeping genes has often been inconsistent [22]. It has also been a great challenge for researchers to distinguish the environmental and the pathogenic strains of *E. cloacae* in previous studies [22]. Phylogenomic analysis was performed using 1732 core genes of eight *Enterobacter* *spp.* and three *Pantoea* *spp* . The four functionally distinctive strains of *E. cloacae* (the plant growth promoting *E. cloacae* subsp. *cloacae* ENHKU01 (ENHKU01) ,the plant pathogen *E. cloacae* EcWSU1 (EcWSU1), the opportunistic human pathogen *E. cloacae* subsp. *cloacae* ATCC13047 (ATCC13047) and the 2,3-butanediol producing *E. cloacae* subsp. *dissolvens* SDM (SDM)) were clustered together, and this observation is consistent with previous studies of the phylogenetic relationships of *E. cloacae* [22](Figure 1).

### General properties of the *E. cloacae* genomes

**Figure 2:**
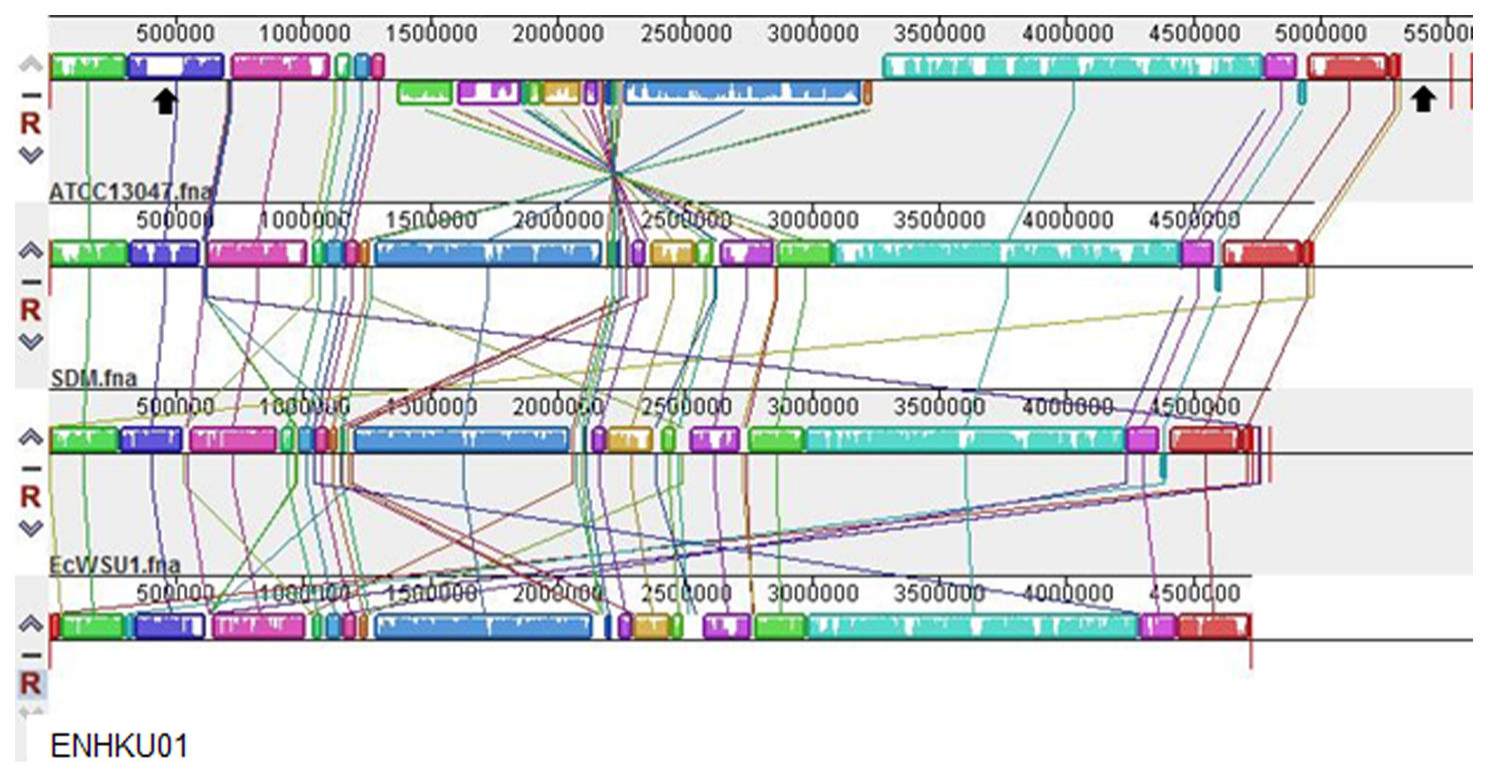
Genomic alignment of *Enterobacter cloacae*. MAUVE [35] alignment of the genome sequences of *E. cloacae* subsp. *cloacae* ATCC13047, *E. cloacae* subsp. *dissolvens* SDM, *E. cloacae* EcWSU1 and *E. cloacae* subsp. *cloacae* ENHKU01. Same color boxes represent homologous regions of sequence, without rearrangement (locally collinear blocks or LCB), shared between *E. cloacae* genomes. Black arrows show the genomic position of the T4SS located in ATCC13047.

The general genome properties of ENHKU01, EcWSU1, ATCC13047 and SDM are shown in (Table 1). The genome of *E. cloacae* subsp. *cloacae* ENHKU01 is composed solely of a single 4.72-Mbp chromosome, and a total of 4338 protein coding regions (CDS) were predicted, with 87% being connected to Clusters of Orthologous Groups (COGs). The genome size, total number of genes and predicted CDS of ENHKU01 is slightly smaller but similar to that of EcWSU1 and SDM, while ATCC13047 appears to have a larger genome of ~5.31 Mb. The genome expansion of ATCC13047 is due to the addition of two plasmids and more than 20 genomic variable regions in its chromosome. EcWSU01 contains one plasmid, and SDM lacks plasmids. There is an absence of sequence similarity between the plasmids in different strains of *E. cloacae*. The genomic GC content of the strains ranges between 54.5-55.1%. MAUVE analysis showed an overall collinear relationship across *E. cloacae* strains, despite a large scale chromosomal reorganization that occurred in ATCC13047 that was conferred by a single recombination event and resulted in the inversion of a genomic region (Figure 2) [35].

**Table 1:** Summary of genome sequence projects of *E. cloacae*.

**Strain**	***E. cloacae* subsp. *cloacae* ENHKU01**	***E. cloacae* subsp. *cloacae* ATCC13047**	***E. cloacae* EcWSU1**	***E. cloacae* subsp. *dissolvens* SDM**
Size	4.73	5.6	4.8	4.97
No. of Chromosome	1	1	1	1
No. of Plasmid	0	2	1	0
GC content %	55.1	54.6	54.5	55.1
Total genes	4445	5639	4740	4646
Predicted CDS	4338	5518	4619	4542
No. of tRNAs	82	24	83	53
No. of rRNA operons	8	8	8	3
Host	Plant	Human	Plant	Soil
Important feature	Endophyte	Human opportunistic pathogen	Plant pathogen	2,3-butanediol production

**Figure 3:**
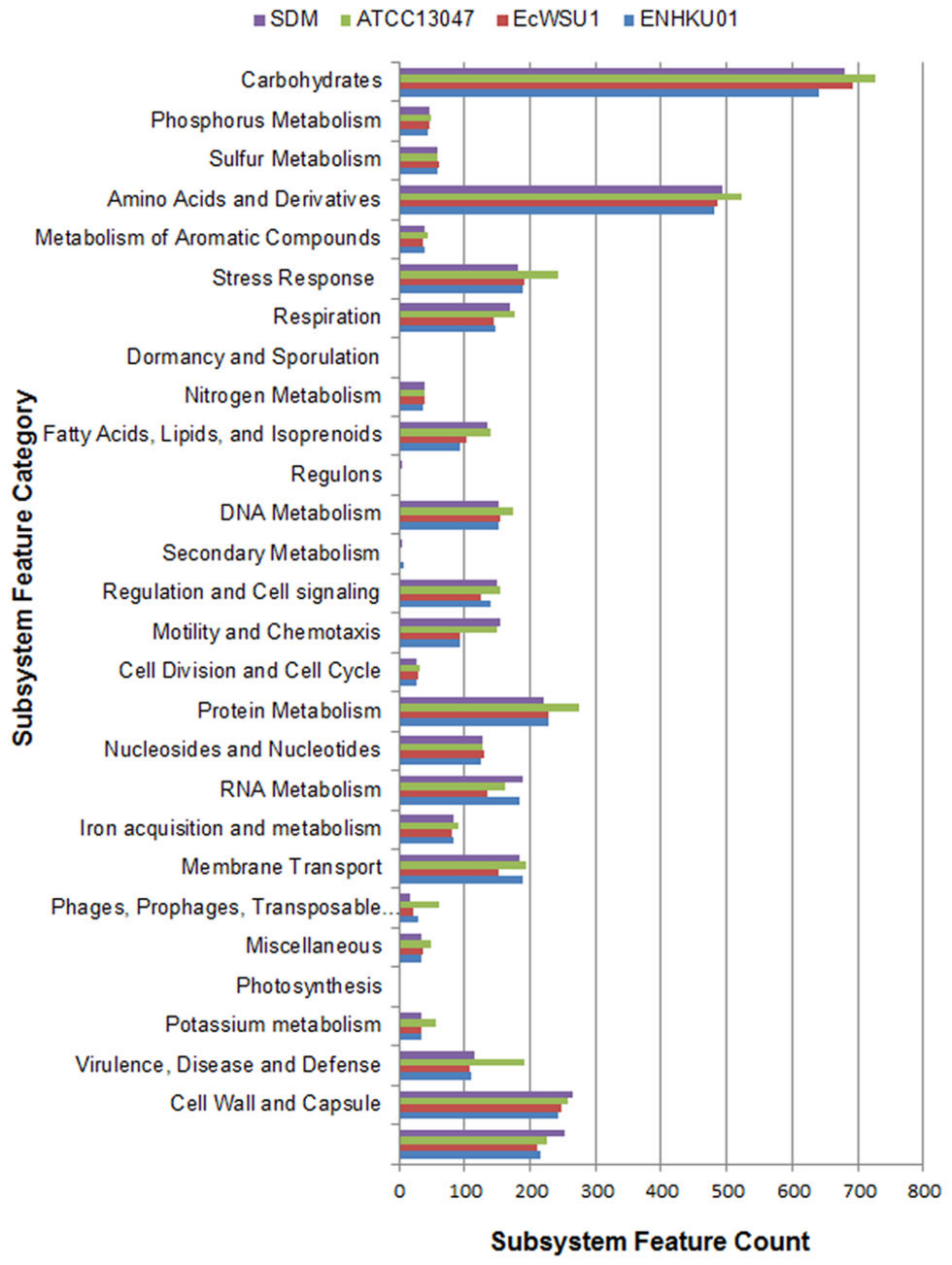
Comparison of subsystem features between *E. cloacae*. Genome sequences of ATCC13047, SDM, EcWSU1 and ENHKU01 were uploaded to the SEED Viewer server (http://rast.nmpdr.org/seedviewer.cgi) independently. Functional roles of RAST annotated genes were assigned and grouped in subsystem feature categories as shown in the figure [26], and colored bars indicate the number of genes assigned to each category. Details for subsystem and functional role assignment for the genes of each strain are listed in Data S1.

Major subsystems and metabolic pathways are conserved between *E. cloacae* strains; however, the number of genes is increased in ATCC13047 in several functional categories compared to other *E. cloacae* strains (Figure 3). *E. cloacae* strains, similar to other *Enterobacter* , are characterized by their ability to use a wide range of carbon sources through their diverse carbohydrate metabolic pathways and transport systems [18]. Over 640 of annotated genes, accounting for 13-15% of the *E. cloacae* genomes, had a designated role for carbohydrate utilization (Figure 3; Data S1), this number is comparable to that of the related genomes in the family of Enterobacteriacae [36,37,38].

**Figure 4:**
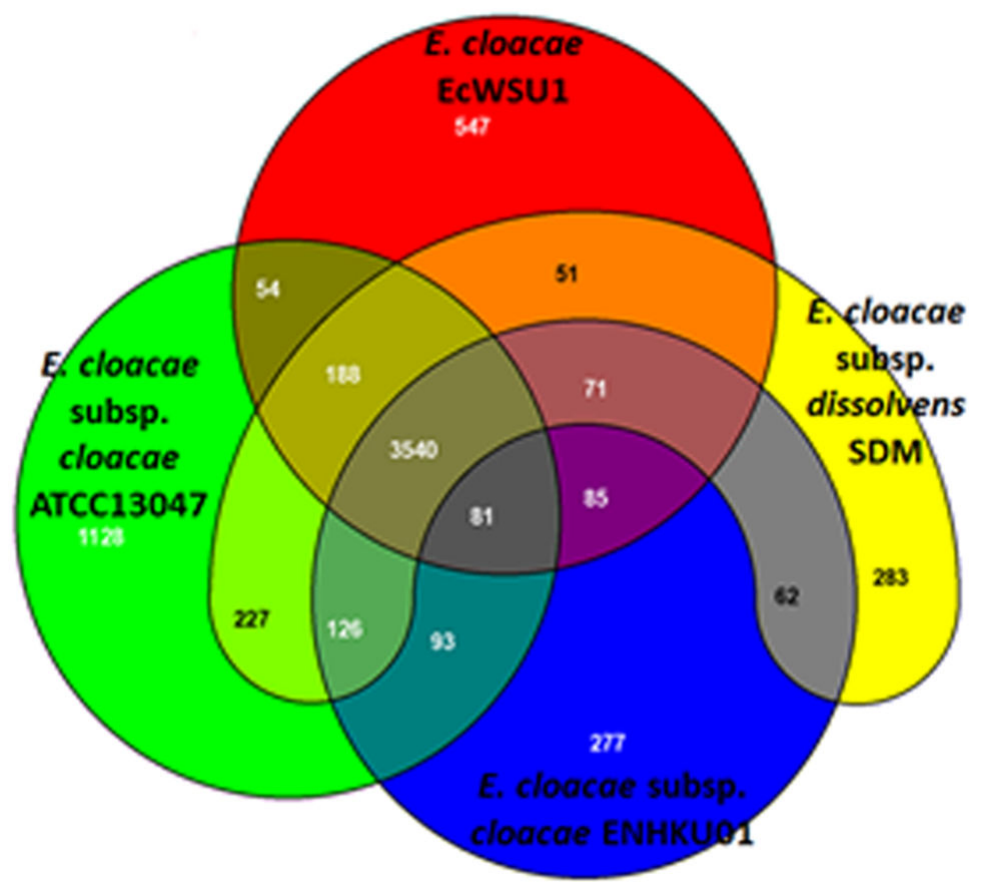
Venn diagram of four *E. cloacae* strains. The Venn diagram shows the pan-genome of ATCC13047, SDM, EcWSU1 and ENHKU01 generated using EDGAR [27]. Overlapped regions represent common CDS shared between the *E. cloacae* genomes. The number outside the overlapped regions indicates the number of CDS in each genome without homologs in other sequenced *E. cloacae* genomes.

Pan-genome analysis was performed for eight *Enterobacter* species, and a further comparison was performed for *E. cloacae* strains (Table S1). As shown in the Venn diagram (Figure 4; Data S2), the four *E. cloacae* strains shared 3540 CDS in their core genome, corresponding to approximately 64%-82% of all CDS in these genomes. A relatively small portion of CDS were shared between two or three *E. cloacae*. Both ENHKU01 and SDM had approximately 6% of unique CDS that are absent in the other *E. cloacae* genomes evaluated, and the unique CDS percentage was 12% and 20% for EcWSU1 and ATCC13047, respectively. The majority of singletons were mostly associated with hypothetical proteins (Data S3), and this observation is consistent with singleton analysis of other species [39,40]. However, a larger number of functional singletons, most likely contributing to virulence, were identified in ATCC13047 (File 4).

### Virulence associated genes

The virulence genes of pathogenic bacteria are often associated with pathogenicity islands that encode a Type III secretion system (T3SS) or a Type IV secretion system (T4SS) acquired by horizontal gene transfer [41,42]. These genomic factors related to pathogenesis and virulence were identified in the plasmid and variable regions in the opportunistic human pathogen *E. cloacae* subsp. *cloacae* ATCC13047, and they are absent in the other *E. cloacae* strains. Two clusters of T4SS genes were found in the plasmid pECL_A and a 139-kb variable region of ATCC13047 in the chromosome, respectively (Figure 2; Table S1). T4SS is associated with pathogenesis in plants and mammalian bacterial pathogens and contributes to genome plasticity in bacteria [43,44]. DNA, effector proteins and virulence factors are delivered to the target host cells by the conjugative transfer machine, although several non-pathogenic strains also carry the membrane transfer system [43,45]. The plasmid pECL_A also contains multiple heavy metal resistance operons for copper, tellurium and mercury that are not conserved with other *Enterobacter* species but share notably high homology to *Cronobacter* *sakazakii* , *Klebseilla* *pneumoniae* and *Escherichia coli* (data not shown), which are all species commonly known as human pathogens or opportunistic pathogens. ATCC13047 is believed to acquire multiple heavy metal resistance genes horizontally from other human microflora associated bacteria, thereby contributing to the adaptation and fitness of the bacteria in heavy metal-rich environments, such as sewage [15].

The pathogenesis of many plant pathogens often involves T3SS and its associated effector proteins [46]. Although *E. cloacae* EcWSU1 was reported to be a plant pathogen that causes *Enterobacter* bulb decay in the onion, no T3SS or T4SS was observed in either the plasmid or the chromosome. It remains largely unclear how EcWSU1 became a plant pathogen.

### Diversity of fimbriae contributes to variation in colonization and host determination of different *E. cloacae* strains

Adhesion to and colonization of the host is one of the key factors for host determination for symbiotic and endophytic bacteria and determining the success of pathogenesis for pathogenic bacteria [47,48]. Fimbriae, also known as pili, are widely distributed among the Proteobacteria and are known to be critical in adhesion and the specific binding to tissues of preferred hosts [49]. Located in the outer membrane, fimbriae subunits are usually assembled into filament structures using the chaperone/usher pathway [47]. We compared the fimbriae in different strains of *E. cloacae* and observed significant diversity. Nine to thirteen fimbrial protein-encoding loci were identified in each strain, but only four of them were conserved across all four *E. cloacae* strains (Table 2; Table S1). ATCC13047 and EcWSU1 each had two unique fimbrial clusters, and four of the ENHKU01 fimbrial clusters are absent in other *E. cloacae* (Table 2; File 2). The presence of multiple fimbriae genes indicates that *E. cloacae* are likely able to colonize a rather wide range of hosts or environments. Variation in fimbriae between different strains of *E. cloacae* is expected to alter the choice of hosts or environmental niches that *E. cloacae* strain can colonize and thus to contribute to the diversity of the species.

**Table 2:** Comparison of fimbriae in *E. cloacae*.

		*E. cloacae* strain			
Pan genome position	Representing Locus	ECENHK	EcWSU1	ATCC13047	SDM
28-31	ECENHK_00140-00155	x			
625-629	ECENHK_03165-03185	x			x
662-667	ECENHK_03350-03375	x	x		
809-815	ECENHK_04095-04125	x		x	x
827-830	ECENHK_04185-04200	x			
1096-1104	ECENHK_05530-05570	x	x	x	x
1377-1381	ECENHK_06955-06975	x	x	x	x
1691-1696	ECENHK_08555-08580	x			
1842-1846	ECENHK_09330-09350	x	x	x	x
2718-2721	ECENHK_13830-13845	x	x	x	x
3633-3636	ECENHK_18495-18510	x			
3728-3731	ECENHK_18970-18985	x		x	x
3900-3903	ECENHK_19830-19845	x	x	x	
4417-4420	ECL_04371-4368			x	
4512-4516	ECL_01105-01109			x	
4918-4922	ECL_03396-03400		x	x	
5476-5479	EcWSU1_00874-00877		x		
5612-5616	EcWSU1_02030-02034		x		
6068-6072	ECL_00070-00074			x	x
6079-6083	ECL_00089-00093			x	x
6118-6122	ECL_00370-00374			x	x
	Total number of fimbriae	13	9	13	10

One of the fimbrial loci in ATCC13047 and SDM, encoding the Colonization Factor Antigen I (CFA/I) fimbrial proteins (ECL_00070-00074), has drawn our attention. The locus is located in a cluster of genes encoding the virulence related resistance-nodulation-division (RND) efflux system within a variable region that is absent in the plant-associated ENHKU01 and EcWSU1strains [50]. CFA/I fimbriae, as a member of alpha-fimbriae belonging to Class 5 fimbriae, have been shown to play a critical role in colonizing the epithelia of the human intestine in the enterotoxigenic strains of *Escherichia coli* (ETEC) [51]. BLAST and alignment analyses further demonstrated that the fimbria are highly associated with human-related *Enterobacter* species [52] (Figure S1), but they are rarely found in plant-associated strains. The *Enterobacter* CFA/I fimbriae is most closely related to *Escherichia coli* (Figure S1), which illustrates that the CFA/I fimbrial gene cluster is a viable candidate for facilitating *Enterobacter* adhesion and colonization in humans. Such a specific correlation was not observed in the other fimbriae found in *E. cloacae*.

### Antagonistic potential of *E. cloacae*: Microbial competition for resources

Microbes compete with each other for limited resources within their communities [53,54]. Certain microbes, primarily demonstrated for *Pseudomonas* spp., compete with other microbes for acquiring ferric ion (iron), an essential growth element from soil. These microbes produce higher affinity siderophores and indirectly suppress the growth of competing fungi, which produce lower affinity fungal siderophores, and non-siderophore producing bacteria [55,56]. Resource competition mechanisms thus lead to antagonistic effects in the rhizosphere. A conserved pathway for enterobacin synthesis was found across *Enterobacter* spp. In addition, *E. cloacae* strains possess an extra siderophore assembly kit for aerobactin siderophore biosynthesis, which is absent in certain other *Enterobacter* spp. (Table 3; Table S1).

**Table 3:** Key genes involved in potential antagonistic activities in *Enterobacter* .

			*E. cloacae* strains				*Other Enterobacter*			
**Pan genome position**	**Description**	**Representing locus**	ENHKU01	EcWSU1	ATCC13047	SDM	LF7a	KCTC 2190	SCF1	638
**Siderophores**										
2810-2814	Aerobactin siderophore biosynthesis	ECENHK_14300-14320	+	+	+	+	-	-	-	-
1196-1208	Enterobactin synthesis	ECENHK_06040-06100	+	+	+	+	+	+	+	+
**Linoleic acid metabolic pathway**										
4185	Lysophospholipase L2	ECENHK_21265	+	+	+	+	+	+	+	+
4189	Phospholipase A	ECENHK_21285	+	+	+	+	+	+	+	+
**Type II secretion system**										
1457-1468	Type II secretion pathway (General secretion pathway)	ECENHK_07365-07425	+	+	+	+	+	-	+	-
**Chitinase**										
1470	Chitinase/ glycoside hydrolase family protein	ECENHK_07430	+	+	+	+	+	-	+	-
1472	Chitinase	ECENHK_07440	+	+	+	+	+	-	+	-
1763	Chitinase/ glycoside hydrolase family protein	ECENHK_08915	+	+	+	+	+	+	-	-
**Colicin V and Bacteriocin Production**										
387-388	Entericidin A/ B	ECENHK_01955-01960	+	+	+	+	+	-	+	+
570	S-type Pyocin domain-containing protein	ECENHK_02880	+	-	+	+	+	-	-	-
681-684	Tolerance to Colicin E2	ECENHK_03445-03460	+	+	+	+	+	+	+	+
3073-3078	Colicin V production protein & bacteriocin production cluster	ECENHK_15640-156470	+	+	+	+	+	+	+	+

Other resources subjected to microbial competition include fatty acids. Earlier functional studies demonstrated that *E. cloacae* competes for the seed exudate linoleic acid against *P* *. ultimum* , which suppresses sporangium germination of the oomycete pathogen and causes the damping-off of seedlings in many crop plants [57]. In this study, we have identified two candidate genes in the linoleic acid metabolic pathway that could play an important role in fatty acid competition. Phospholipase A1 Precursor (E.C. 3.1.1.4) and Lysophospholipase L2 (EC 3.1.1.5) are expected to hydrolyze lecithin (Table 3; File 2), which is an exudate lipid molecule composed of choline, linoleic acid, phosphorus and inositol, and initiate the fungal/ oomycete responses of seeds. Both genes are located adjunct to a multiple sugar transporter and are found across different *Enterobacter* genomes [57].

### Chitinases

**Figure 5:**
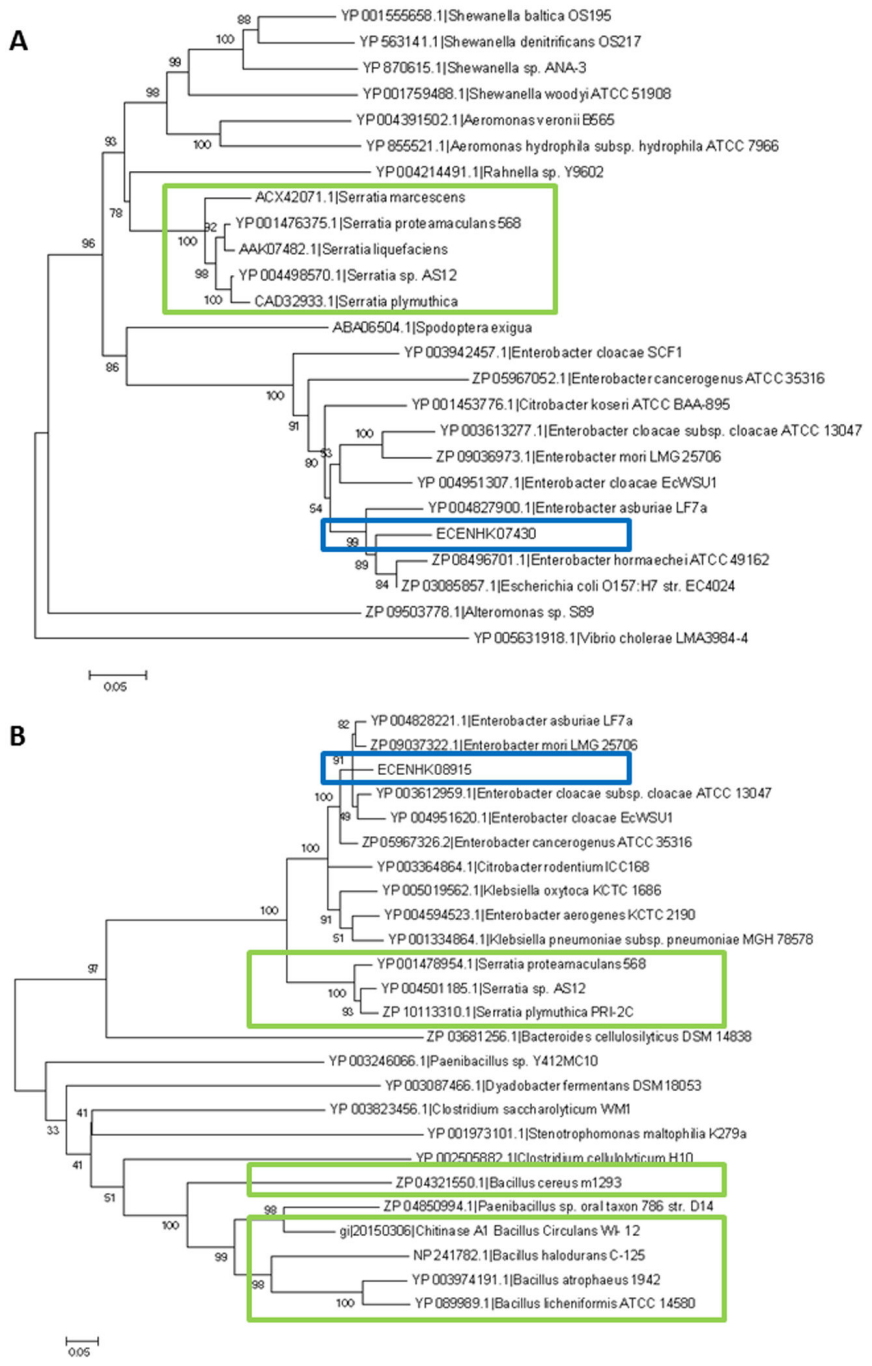
Phylogenetic analysis of chitinase genes. Neighbor-joining tree with bootstrap values of chitinase genes associated with (A) ECENHK_07430 and (B) ECENHK_08915 constructed by twenty-five representing orthologs from each species using MEGA5. The blue box indicates the corresponding chitinase gene of ENHKU01. Chitinases that have been functionally characterized for their antifungal activities are highlighted in green boxes [87,88,89,90,91].

The ability of microbes to produce a wide range of antimicrobial compounds, including lytic agents, antibiotics, bacteriocins, protein exotoxins and other secondary metabolites, is critical to their success in antagonistic activities. In bacterial-fungal interactions, cell wall hydrolases, such as certain proteases and chitinases, are produced and secreted extracellularly by bacteria to target fungal cell walls [58,59,60]. Chitinases are lytic enzymes that break down glycosidic bonds in chitin, a major component of fungal cell walls, and have been demonstrated to effectively inhibit fungal growth. Three conserved putative chitinase genes have been identified across *E. cloacae* genomes. Two of these genes are located adjacent to a type II secretion system [61], while the third one is located elsewhere (Table 3; Table S1). The Type II secretion system and the associated chitinases are absent in *Enterobacter* sp. 638 and *E* *. lignolyticus* SCF1. Phylogenetic analysis has shown that ECENHK_07430 and the synthetic *Enterobacter* chitinases are associated with the well-characterized *Chitinase A* in *Serratia marcescens* [62,63] (Figure 5), which has been demonstrated to control several important plant pathogenic fungi including *Botrytis* spp., *Rhizoctonia solani, Fusarium oxysporum* f. sp. *cyclaminis* and *Sclerotinia* spp. In fact, a functional *Chitinase A* that effectively controls *Rhizoctonia solani* has been identified in *E. agglomerans* [64]. The other two chitinases have not been reported in any *E. cloacae* isolates. Orthologs of ECENHK_08915, can also be found in several related species of *Klebsiella* , *Citrobacter* and *Serretia*, but it is absent in other family members of Enterobacteriaceae. Interestingly, phylogenetic analysis further demonstrated the association between the *Enterobacter* ’s chitinase and *Chitinase A1* of the gram-positive *Bacillus* spp. (Figure 5). It is notable that *Bacillus* spp. are well-known as plant growth-promoting rhizobacteria (PGPR), as they have antagonistic effects against other microorganisms and are used commercially as biological control agents for various fungal pathogens [65,66].

### Production of other antimicrobial compounds

Several bacteria and fungi exhibit antagonistic effects in microbial competition through the production of antibiotics [67]. In microbial-microbial competition, other bacteria also develop detoxification mechanisms and/or antibiotic resistance to counteract anti-microbial compounds. Previous genome analysis demonstrated *Klebsiella* and *Enterobacter* possess a number of antibiotic resistance genes and multidrug efflux systems [37,68], these multiple antimicrobial mechanisms were also observed in *E. cloacae* (Table S2). One can expect, due to the wide range of antimicrobial resistance, multidrug resistance proteins and multidrug efflux systems benefit the survival of *E. cloacae* against both bacterial and fungal competitors in habitats with high microbial competition, and furthermore, this phenomenon explains how clinical strains of *E. cloacae* raise medical concerns [13,14].

The production of bacteriocins also plays an important role, particularly in bacteria-bacteria interactions [69]. Well-characterized mechanisms in gram-negative bacteria include the enteric bacteriocin, colicin [70] and the lipoprotein Entericidin [71]. Antimicrobial compounds identified in *E. cloacae* that are related to bacteriocin production involve the Colicin V and bacteriocin production cluster [69], tolerance to colicinE2, Entericidin A and B [71] and S-type Pyocin (Table 3; Table S1). Additionally, other bacteriocin production-related genes were found to be associated with the Type VI secretion system (T6SS) in *E. cloacae.*

### Type VI secretion systems and their roles in microbial competition

**Figure 6:**
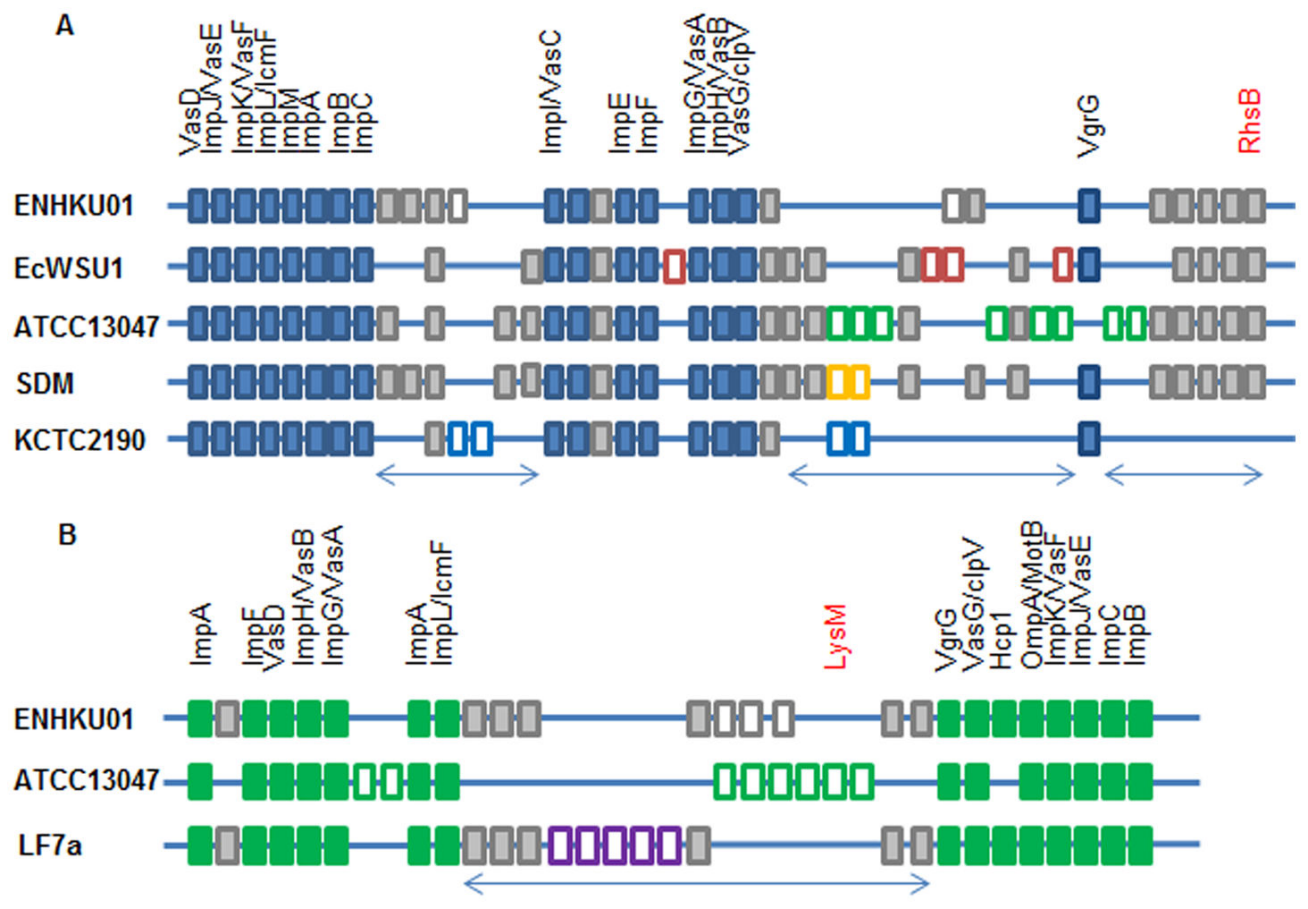
Genetic organization of T6SS in *Enterobacter* . (A) and (B) show genetic organization of the two T6SS clusters commonly found in *Enterobacter* . ENHKU01 and ATCC13047 contain both T6SS, and EcWSU1, SDM and LF7a have one of the two, and other *Enterobacter* have none. The two clusters of T6SS in *Enterobacter* have different genetic organization but are aligned across the *Enterobacter* genomes at the corresponding loci. Each T6SS cluster is composed of conserved regions formed by conserved T6SS core component genes, which are indicated in solid blue/ green color boxes, and variable regions that are indicated by arrows. The variable regions contain a variable number of conserved genes (solid gray color) and unique genes (white color boxes). Most genes located in the variable regions are described as hypothetical proteins. Genes possibly involved in bacteriocin activity are shown in red. Details of the genetic organization of T6SSs in *Enterobacter* are listed in Data S4.

The T6SS is widely distributed in gram-negative bacteria. A T6SS usually involves approximately 15 conserved genes in a cluster with genes encoding a functional secretory apparatus that penetrates cell membranes and translocates effector proteins into their eukaryotic hosts or recipient cells [72,73]. Earlier studies have related the T6SS to virulence in humans and animals [74]; however, recent studies have demonstrated the directed function of the T6SS toward microbial interaction and fitness in microflora [75,76,77,78]. It is common to find more than one cluster of T6SS genes within a genome [32,79,80]; both ATCC13047 and ENHKU01 contain two clusters of T6SS, while SDM and EcWSU1 have only one. Each T6SS gene cluster is composed of sets of conserved core component genes and variable regions distinguished by hypothetical proteins (Figure 6). A recent discovery allowed us to understand more about the roles of these hypothetical proteins in microbial competition as anti-bacterial effectors. Genetics and characterization of the bacteriolytic effectors *Tse1*, *Tse2* and *Tse3* in *Psuedomonas* *aeruginosa* revealed that T6SS effector proteins target the peptidoglycan of prokaryotic cells [76]. Within the variable region of *E. cloacae* T6SS, we found RhsB (a rhs-like genetic element involved in bacteriocin production) [81,82,83] and LysM (involved in bacterial cell wall degradation) [84,85] (Figure 6). It is believed that bacteria deliver different effector proteins to their specific target counterparts among the microflora using a conserved T6SS apparatus [76,83].

**Figure 7:**
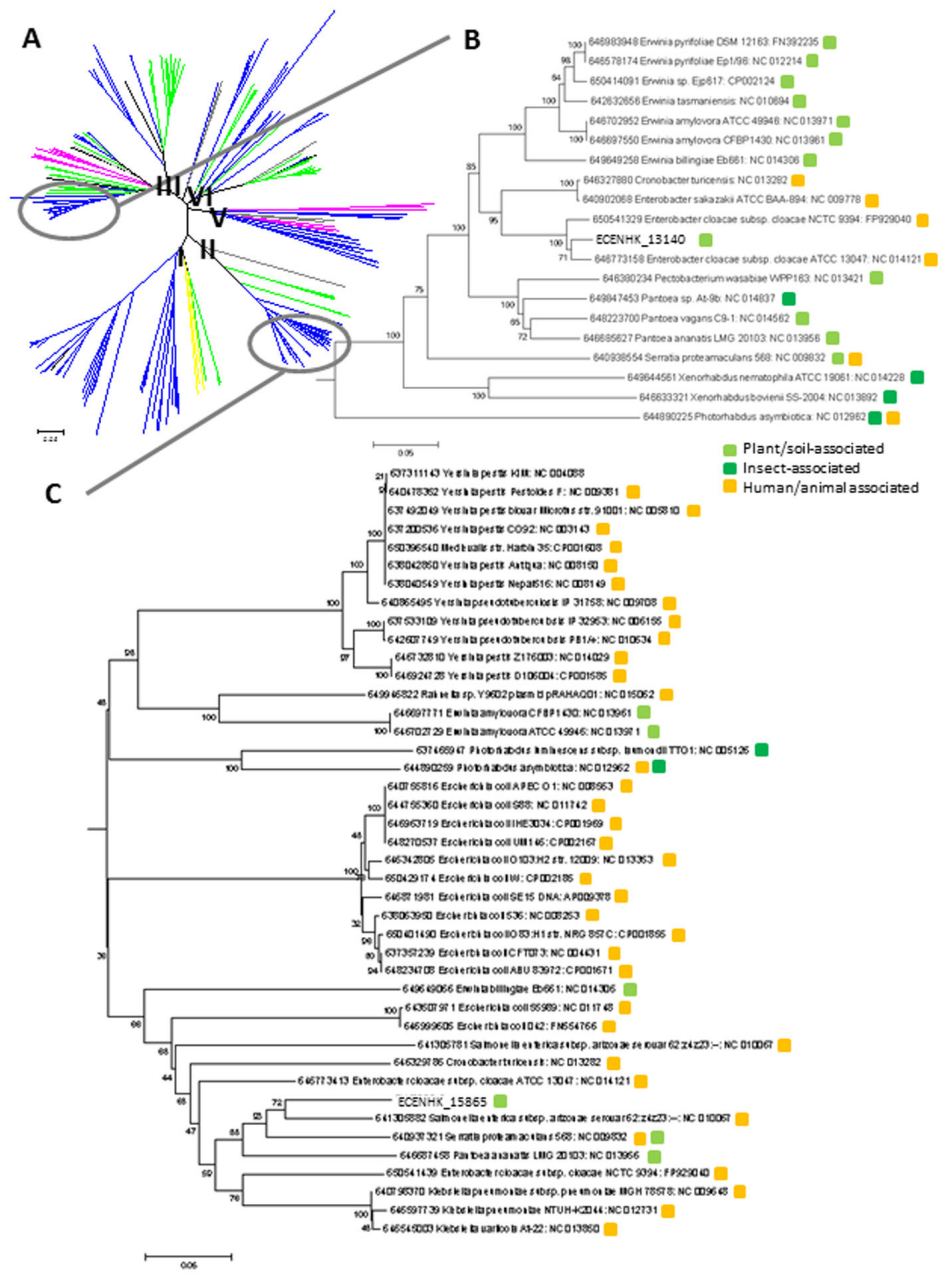
Phylogenetic analysis of ClpV. (A) The neighbor-joining tree of T6SSs using ClpV orthologs from 348 T6SS clusters in 231 species. The 231 species are grouped according to their class, which is indicated using color lines: α for the subdivision of Proteobacteria (Pink), β subdivision (Green), γ subdivision (Blue), δ/α subdivision (Black) and other bacteria unrelated to Proteobacteria (Gray). ClpV orthologs are distributed in five clades and named I-V. Naming of clades is according to Boyer et al [86]. Each clade is contributed by different bacterial families of Proteobacteria. Our result is consistent with a previous phylogenetic analysis of T6SS using 13 T6SS conserved component genes [86]. ClpV of *E. cloacae* are distributed in clades II and III of the phylogenetic tree and are clustered together with other strains and species in the family of Enterobacteriaceae possessing T6SS, thus forming sub-trees in each clade (indicated by gray circles). A simplified and enlarged version of the neighbor-joining tree with bootstrap values showing (B) the sub-tree of clade III formed by ClpV orthologs associated with ECENHK_13140 and (C) the sub-tree of clade II associated with ECENHK_15865. Color squares indicate the habitats of the corresponding bacterial species: plant/ soil-associated (light green), insect-associated (green) and Human/ animal associated (orange).

The sequences and genetic organization of core component genes in each T6SS cluster are aligned across the genomes of *Enterobacter* , but the T6SS clusters within a genome are not associated with one another (Figure 6; Data S4). There is also variation in the core component genes of each cluster (Figure 6). Such differentiation indicates that these T6SSs are not only evolutionarily distinct but also functionally and structurally different from each other [32]. Phylogenetic analysis of the representative T6SS component gene ortholog, ClpV, indicated that two evolutionary distinct T6SSs were introduced into *E. cloacae* independently before divergence, as ClpV orthologs of *E. cloacae* are distributed in two clades (Clade II and Clade III) (Figure 7A). In clade III, orthologs are clustered with those found in other plant and soil-associated bacteria of Enterobacteriaceae, such as the plant pathogens *Erwinia* *amylovora* and *Pectobacteria* *wasabiae* , and soil-borne bacteria, such as *Pantoea* *vagans* C9-1 and *Erwinia* *pyrifoliae* Ep1/96 (Figure 7B); also, there are many human and animal pathogenic/associated bacteria in this family, such as *Escherichia coli*, *Klebsiella pneumoniae*, *Yersinia pestis* are located in Clade II (Figure 7C). Our observation of the correlation between the phylogeny of T6SSs and the ecological niches of bacteria is in line with a previous T6SS phylogenetic analysis study [86].

The presence of T6SS in *E. cloacae* strains is expected to allow the bacterial strain to enhance the survival of non-pathogenic strains in their habitats, thus competing with other microbes for the colonization of hosts [75]. *E. cloacae* strains possessing more than one distinct T6SS cluster, such as ATCC13047 and ENHKU01, are likely to have fitness advantages in a borader range of habitats and be able to survive in different environments.

### Antagonistic activities of *E. cloacae* subsp. *cloacae* ENHKU01

**Figure 8:**
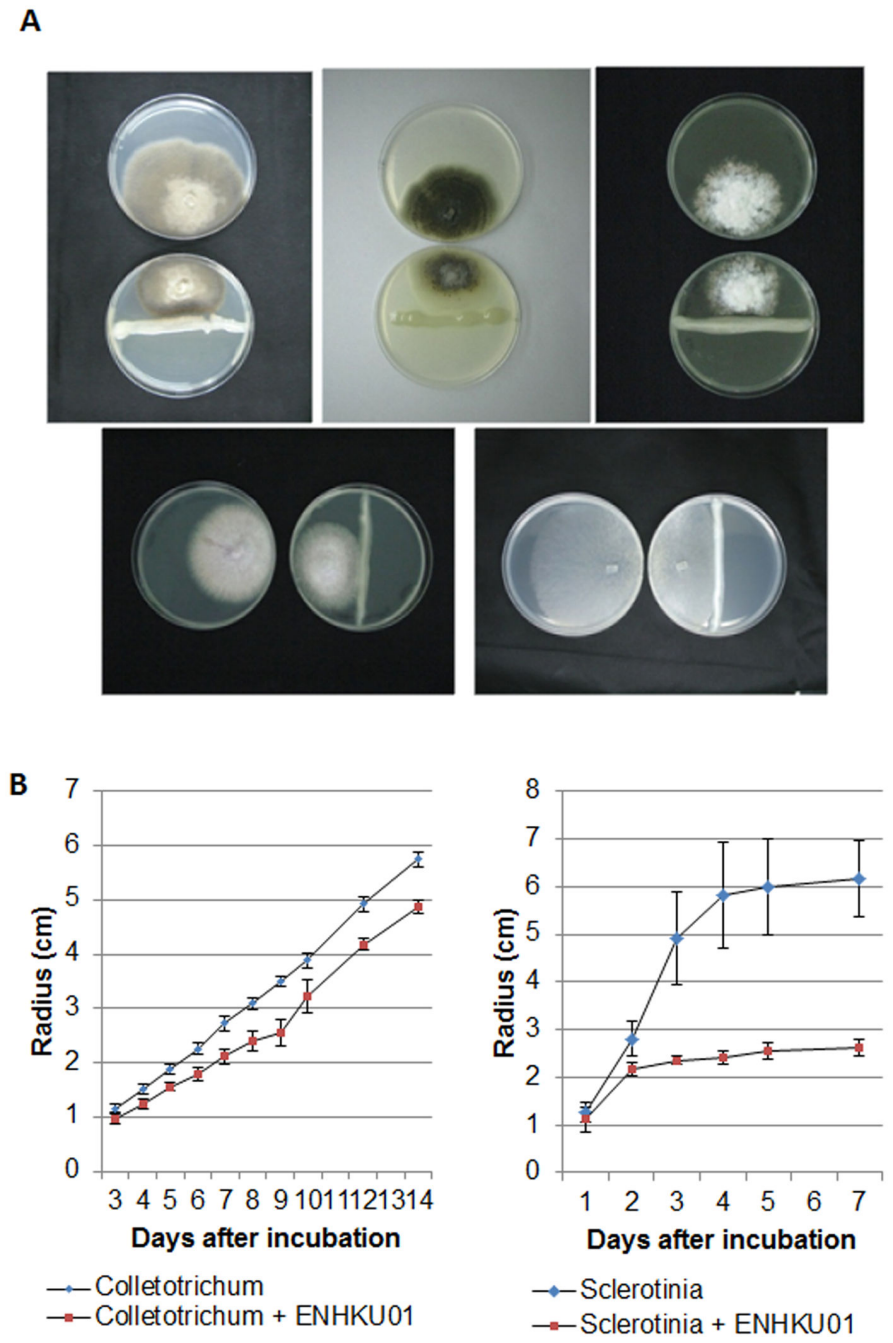
Antagonistic activity of *E. cloacae* subsp. *cloacae* ENHKU01 against fungi. (A) Visualization of fungal growth with and without ENHKU01: (from left to right, upper row) *Alternaria* sp., *Colletotrichum* *capsici* , *Didymellabryoniae* , (from left to right, lower row) *Fusarium oxysporum* and *Sclerotinia sclerotiorum*. Photos were taken 7 days after incubation; (B) Growth of *Colletotrichum* *capsici* (Col) and *Sclerotiniascleotiorum* (Scl) were closely monitored with and without ENHKU01. Challenging fungi were grown on PDA plates as described in Methods and Materials, the radius of growth of hyphae (in cm) was measured. Numbers show an average of 10 plates, and error bars represent the S.D. from the mean.

**Figure 9:**
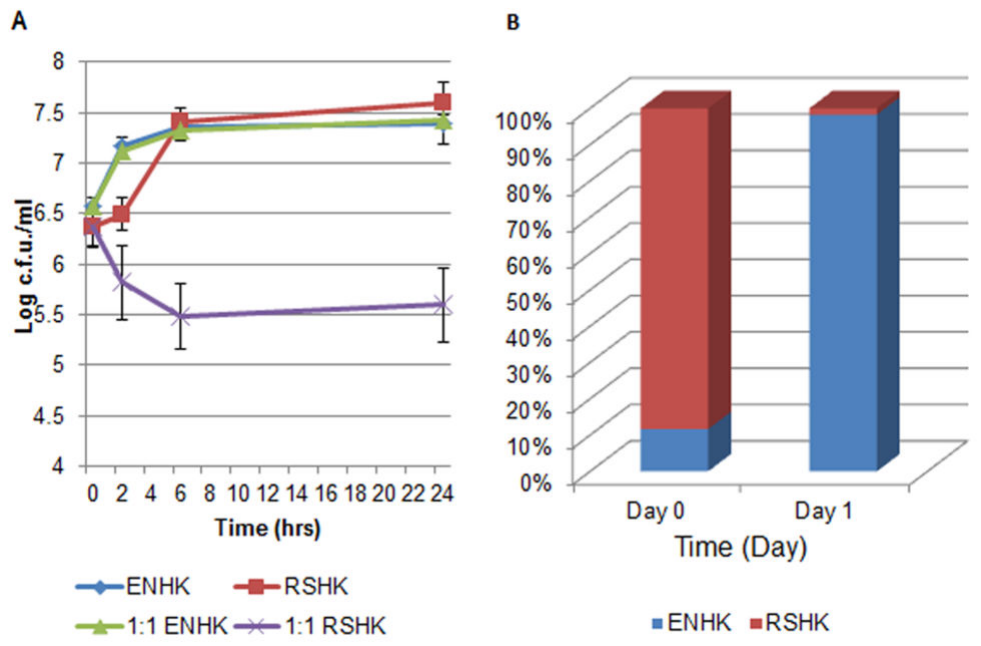
Antagonistic activity of *E. cloacae* subsp. *cloacae* ENHKU01 against *R. solanacearum*. Figures show results of ENHKU01 – *R. solanacearum* competition assays: (A) the planktonic culture with the quantities of *E. cloacae* subsp. *cloacae* ENHKU01 (ENHK) and *R. solanacearum* (RSHK) in log C.F.U. per ml recorded at 0, 2, 4, 6 and 24 hours after incubation. Numbers show an average of 4 replications, and error bars represent the S.D. from the mean; (B) Biofilm culture, relative percentage of *E. cloacae* subsp. *cloacae* ENHKU01 (ENHK): *R. solanacearum* (RSHK) at day 0 and day 1 of incubation.

Through genome analysis, the antagonistic potential of *E. cloacae* has been revealed in this study, and we further hypothesized that *E. cloacae* has advantages in microbial competition against other microbes within its environmental niche. To investigate the effect of antagonistic activities in *E. cloacae* strains further, we performed bacterial-fungal and bacterial-bacterial antagonistic assays using the plant growth-promoting endophytic ENHKU01 as a model. As expected, ENHKU01 showed significant antagonistic effects against a wide range of fungal species: *Collectotricumcapsici* . *Sclerotinia sclerotiorum*, *Alternaria* sp., *Didymellabryoniae* , and *Fusarium oxysporum* by suppressing their growth at different levels (Figure 8; Table S3). These fungal species are common plant pathogens, and *C* *. capsici* and *S. sclerotiorum* are direct competitors with ENHKU01, sharing the pepper plant as a host. Additionally, in our bacteria-bacteria competition assay, it was demonstrated that ENHKU01 effectively suppressed the growth of *Ralstonia solanacearum*, a devastating bacterial pathogen with a wide host range that is most well-known for causing bacterial wilt in Solanaceous crops, such as tomato, pepper and eggplant (Figure 9). Taken together, these bioassays provide further evidence, drawn from comparative genome analysis, demonstrating the antagonistic potential of *E. cloacae*.

### Conclusions

Comparative genome analysis reveals the antagonistic potential of *E. cloacae*. The multiple antagonistic mechanisms of *E. cloacae* are expected to contribute its success in competition against other microbes in various environmental niches, thus allowing different strains of *E. cloacae* to survive in diverse environments.

## Supporting Information

Data S1 The functional roles of RAST annotated genes were curated into subsystems by SEED Viewer, and the details are listed in this file. The numbers of genes grouped in each subsystem feature category for each *E. cloacae* strain were counted as shown in the summary page.
(XLSX)

Data S2 A Venn diagram of the data generated by EDGAR [27].
(XLSX)

Data S3 List of singletons of ENHKU01, EcWSU1, ATCC13047 and SDM generated by comparing the genomes of the four of *E. cloacae* strains using *E. cloacae* subsp. *cloacae* ENHKU01 as the reference genome [27].
(XLSX)

Data S4 List of genes associated with Type VI Secretion Systems in *Enterobacter* , corresponding to Figure 6A and Figure 6B.
(XLSX)

Figure S1 The comparative analysis of the CFA/I Fimbrial cluster.
(DOCX)

Table S1 Pan-genome analysis of *Enterobacter* by EDGAR.In brief, *E. cloacae* subsp. *cloacae* ENHKU01 was selected as reference genome and its gene contents were taken as the base set for stepwise comparison of the seven *Enterobacter* genomes involved in this study. For each round of all-against-all comparison based on a bidirectional best BLAST hit, every gene in a *Enterobacter* genome that has no ortholog in the base set was added to the reference set and compared with the next genome. The process was repeated step by step to obtain the pan-genome of *Enterobacter* [27].
(XLSX)

Table S2 List of genes involved in potential antibiotic resistance, multidrug resistance and multidrug efflux systems in *Enterobacter* .
(XLSX)

Table S3 Table shows antagonistic activities of *E. cloacae* subsp. *cloacae* ENHKU01 against plant pathogenic fungal species.
(DOCX)
