# Supplementary material for: Comparative Genome Analysis of Enterobacter cloacae
Source: PLoS One. 2013 Sep 12;8(9):e74487. doi: 10.1371/journal.pone.0074487 (PMC3771936; doi:10.1371/journal.pone.0074487)

**Comparative analysis of CFA/I Fimbrial cluster**

(A) The variable region of ATCC13047 containing CFA/I Fimbrial component genes and the flanking conserved region is aligned with the corresponding regions in ENHKU01 and displayed by WebACT. Operons encoding for the CFA/I Major subunit (ECL_00070), auxiliary subunit (ECL_00071), usher protein (ECL_00072), minor subunit (ECL_00073) and chaperon protein (ECL_00074) are shown in Black/White arrows. RND efflux system component genes (*eefA*, *eefB*, *eefC*) and the transposable element IS903 are shown in orange and red respectively. (B) Homologs of CFA/I fimbrial component genes were identified by BLASTX and aligned by CLUSTALW. Partial alignment is displayed for the CFA/I usher protein encoding ECL_00072 with its most closely related homologs in *Enterobacter homaechei* ATCC49162, *Enterobacter cancerogenus* ATCC35316 and *Escherichia coli* strains 55989 and O26:H11.


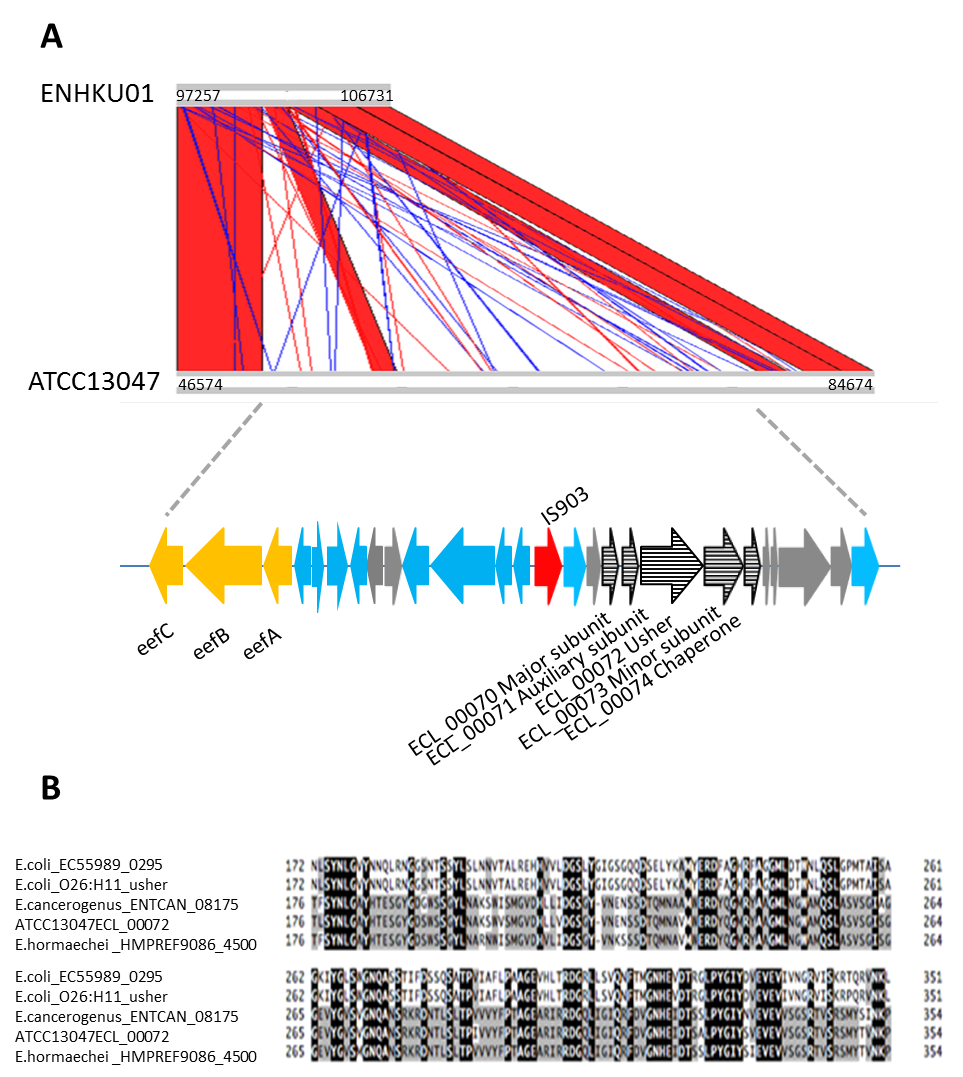

Supplement: Figure S1 — The comparative analysis of the CFA/I Fimbrial cluster. (DOCX) [file pone.0074487.s005.docx]
