# Supplementary material for: Comparative Genome Analysis of Enterobacter cloacae
Source: PLoS One. 2013 Sep 12;8(9):e74487. doi: 10.1371/journal.pone.0074487 (PMC3771936; doi:10.1371/journal.pone.0074487)
Supplement: Table S3 — Table shows antagonistic activities of E. cloacae subsp. cloacae ENHKU01 against plant pathogenic fungal species. (DOCX) [file pone.0074487.s008.docx]

**Antagonistic activities of E. cloacae subsp. cloacae ENHKU01 against plant pathogenic fungal species**

| Species | Disease | Host | Antagonistic effects^ |
| --- | --- | --- | --- |
| *Alternaria sp.* | Alternaria Leaf Spot | Lettuce | +++ |
| *Choanephora infundibulifera* | Choanephora Fruit Rot | Eggplant | - |
| *Colletotrichum capsici* | Anthracnose | Pepper | ++ |
| *Didymella bryoniae* | Gummy Stem Blight | Watermelon | +++ |
| *Fusarium oxysporum* | Fusarium wilt | Cucumber | ++ |
| Sclerotinia *sclerotiorum* | Sclerotinia rot | Pepper | +++ |
| *Sclerotinia* rolfsii | Southern blight | Antirrhinum | +/-* |

^Antagonistic effects of ENHKU01 against fungal growth was rated based on two weeks of observation: “-“ no antagonistic effect; “+” mild antagonistic effect: slightly reduction of mycelium growth; “++” significant reduction of mycelium growth; “+++”growth of mycelium was totally inhibited by ENHKU01. * Mild antagonistic effect was observed during early interaction, growth of mycelium recovered after 7 days.
